# Experience and challenges delivering hepatitis C virus treatment for people who inject drugs in Kenya

**DOI:** 10.3389/fmed.2024.1429516

**Published:** 2024-10-21

**Authors:** Joyce Boke, Aliza Monroe-Wise, Grace Umutesi, Loice Mbogo, Betsy Sambai, David Bukusi, Bhavna Chohan, John Scott, Esther Gitau, William Sinkele, Helgar Musyoki, Joshua Herbeck, Carey Farquhar, Brandon L. Guthrie

**Affiliations:** ^1^Global Assistance Program-Kenya, University of Washington, Nairobi, Kenya; ^2^Department of Global Health, University of Washington, Seattle, WA, United States; ^3^HTC and HIV Care, Kenyatta National Hospital, Nairobi, Kenya; ^4^Department of Medicine, University of Washington, Seattle, WA, United States; ^5^Support for Addiction Prevention and Treatment in Africa (SAPTA), Nairobi, Kenya; ^6^National AIDS and STI Control Programme (NASCOP), Kenyatta Ministry of Health, Nairobi, Kenya; ^7^Department of Epidemiology, University of Washington, Seattle, WA, United States

**Keywords:** hepatitis C virus, people who inject drugs, Kenya, sub-Saharan Africa, HCV treatment

## Abstract

Despite having a higher risk of hepatitis C virus (HCV) infections, people who inject drugs (PWID) in sub-Saharan Africa (SSA) have limited access to HCV treatment. There is scarce literature on treatment delivery modalities that overcome logistical and financial barriers. We utilized different service delivery modalities to provide direct-acting antivirals (DAAs) to PWIDs infected with HCV through methadone clinics and needle and syringe program (NSP) sites in Kenya. In collaboration with Kenya’s National AIDS and STI Control Programme (NASCOP), we enrolled individuals with active HCV infection confirmed by HCV RNA detection from methadone and NSP sites in Nairobi, Mombasa, and Kilifi counties. Liver function and hepatitis B virus (HBV) status were assessed at baseline. Those eligible for treatment were offered ledipasvir-sofosbuvir treatment provided by NASCOP through directly observed therapy (DOT). Participants completed a follow-up visit 12 weeks after completing treatment to measure sustained viral response (SVR-12). Challenges faced while delivering HCV treatment at participating sites included the limited availability and reliability of laboratory assays, and financial constraints faced by PWIDs to attend daily DOT. Based on our experience, strategies to deliver HCV treatment for PWID in Kenya should consider improving the availability of laboratory tests and prioritizing treatment through methadone centers to achieve good outcomes.

## Introduction

Hepatitis C virus (HCV) is a major public health concern with over 71 million people chronically infected globally (1). HCV is responsible for more than half of chronic liver disease cases worldwide (2, 3). To date, no vaccine is available to prevent HCV infections but direct-acting antivirals (DAAs) are highly efficacious treatments with minimal side effects (4–6).

The World Health Organization (WHO) set ambitious goals to eradicate HCV by 2030 through the implementation of high-impact interventions including using DAAs to treat 80% of those who are eligible, reduce the incidence of new infections by 90%, and reduce liver-related mortality by 65% (7). Additionally, targets set by the WHO include improving access to testing and treatment and scaling up harm reduction services (7). However, the cost of treatment, as well as limited access to simpler testing and diagnostic services, have been hurdles to delivering HCV care services in resource-limited settings (8).

People who inject drugs (PWID) are at higher risk for HCV infections than the general population, with an estimated 23–39% of new HCV infections occurring among PWID globally. Additionally, 1 in 3 HCV deaths worldwide is attributable to injecting drug use (1, 9, 10). In Kenya, HCV prevalence among PWID is estimated to be between 18–30%, compared to a general population prevalence of 0.2–1.8% (11–15). Fortunately, in the past 5 years there have been efforts to treat PWID living with HCV in Kenya; however, the number of PWID who have been successfully treated remains low due to financial barriers and limited access to direct acting antivirals and diagnostic tests (16). It is crucial to address this public health concern and provide HCV testing and treatment services for PWID and others with limited access globally and in Kenya to achieve HCV elimination goals. Focusing on this population that accounts for the majority of new infections creates a unique opportunity for micro-elimination, which will serve as a stepping stone to broader HCV elimination efforts in Kenya. To achieve this, targeted approaches are needed to address challenges related to treatment for PWID, including competing needs such as housing, food, withdrawals, comorbidities, poor relations with service providers, and fear of stigma (17–21).

Here we share our experiences and lessons learned in leveraging on existing systems for confirmation of active infection, determining eligibility for treatment, and providing HCV treatment to PWID in 3 counties in Kenya. This project was designed and delivered in collaboration with the Kenyan Ministry of Health (MOH).

## Approach

### Sites selection

The treatment project took place in Nairobi and in Mombasa and Kilifi counties in the coastal area of Kenya. Participants were enrolled and followed up at different public health centers including Methadone treatment centers and needle and syringe programs (NSPs) in Nairobi, Kilifi, and Mombasa counties. NSP centers offer a variety of services including HIV testing, counseling and treatment, needles and syringes, and treatment of minor health conditions. In addition, NSPs also served as referral centers to methadone treatment programs that provide methadone and HIV care and treatment. In Nairobi, HCV treatment activities were conducted at three NSPs operated by Support for Addictions Prevention and Treatment in Africa (SAPTA), a local non-profit organization that offers substance abuse prevention and treatment programs, and at a methadone clinic operated by a health center through the Ministry of Health (MOH). On the coast area, HCV treatments were offered at four NSPs and a methadone clinic located at a sub-county hospital.

### Target population and ethical considerations

The project was nested within the Study of HIV, Hepatitis C, Assisted Partner Services, and Phylogenetics among PWID (SHARP study) (NIH R01-DA043409). The SHARP study was an NIH-funded project that sought to identify, test, and link to HIV care the partners of HIV-positive PWID. Treatment project participants were PWID and their sexual and injection partners enrolled in the parent SHARP study who were diagnosed with active HCV through confirmatory HCV polymerase chain reaction (PCR) testing. Testing for HCV was conducted as part of the study procedures, and individuals found to be HCV antibody positive were referred for evaluation for HCV treatment (22). Recruitment for the HCV treatment project started in November 2020 and the last follow-up visits to confirm HCV cure based on sustained virologic response 12 weeks after treatment (SVR-12) were completed in October 2021. All treatment activities occurred during the COVID-19 pandemic, which was accompanied by periodic travel restrictions, and restrictions in place to minimize in-person contact between patients and clinicians that had some impacts on service delivery in the community. At the time of recruitment, DAAs ledipasvir-sofosbuvir (Harvoni) were provided by the Kenyan Ministry of Health through the National AIDS & STI Control Program (NASCOP). Participants had to meet treatment inclusion criteria to be eligible for treatment (Table 1). The evaluation of the treatment project was approved by the University of Washington (UW) Institutional Review Board (IRB) and the Kenyatta National Hospital (KNH) Ethics and Research Committee. We also obtained approvals from Kenya’s National Commission for Science, Technology, and Innovation (NACOSTI) prior to implementation.

**Table 1:** Treatment inclusion and exclusion criteria.

Inclusion criteria	Exclusion criteria
Positive PCR test for HCV	
Genotype 1 or 4	Active TB infection undergoing treatment
18 years of age or older	Breastfeeding women
PWID population or partner of a PWID	Pregnant women or planning to become pregnant
Willing to provide consent for treatment	Severe uncontrolled psychiatric condition
Willing and able to stay within the catchment area of the treatment site for daily observed therapy (DOT)	Decompensated cirrhosis (Child’s Pugh’s class B or worse) or hepatocellular carcinoma
	Confirmed resistance to Harvoni
	Potential drug–drug interactions

### Designing of treatment delivery models

HCV treatment was delivered through directly observed therapy (DOT) at the methadone clinics and NSPs sites, aligning with treatment protocols developed by MOH/NASCOP. Participants traveled daily to the treatment sites to receive their medication and no travel reimbursement or incentives were provided to align with the standard care delivery processes of these sites. Part of the SHARP study procedures, a small travel reimbursement (~4 USD) was provided at the enrollment and the SVR-12 visit for those who participated in the evaluation study of the treatment project. Participants who were enrolled in methadone programs received their HCV treatment along with their methadone while those not on methadone reported to the nearest NSP site for daily treatment.

### Training of providers

Prior to study implementation, training was conducted for clinical officers from each site who were responsible for overseeing the treatment delivery. These providers were familiar with the participants through their day-to-day interactions at the methadone programs and NSPs. Additionally, they conducted consultative work including clinical assessments, laboratory tests, and dispensed treatment as part of their daily work. Before initiating the delivery of treatment, clinical officers (CO) and other healthcare providers underwent an intensive three-day training, provided by NASCOP and its partners (University of Washington-UW, Kenya Red Cross, and Médecins Sans Frontières -MSF). This training included modules on the basics of HCV (including testing, diagnosis, and treatment), counseling skills, harm reduction services, and how to conduct physical examination for any signs and symptoms of advanced liver disease (including ascites and other stigmata of cirrhosis). This training took place during the second wave of the COVID-19 pandemic in Kenya, which limited in-person meetings, and the training program was held virtually via Zoom. Additionally, refresher training was delivered as needed throughout the treatment process.

### Training of peer educators

Peer educators, who are recovering PWID with established networks in the PWID communities, worked within the treatment sites to improve linkage to care and reduce dropouts. Peer educators are employed at the NSP and methadone centers where they receive monthly stipends of $65 for the treatment project. They were involved in the tracing and follow-ups of identified participants bringing them to the appropriate treatment sites; receiving a reimbursement of $3.50 USD for every participant they brought and followed up successfully. In total, across all sites, 19 peer educators underwent a 1-day training to educate them on HCV, treatment procedures, as well as their roles and expectations during the treatment process.

### Screening and clinical assessment

HCV screening was offered to PWIDs through HCV rapid antibody testing using SD Bioline HCV rapid diagnostic tests (RDT). Individuals with positives results samples were taken for confirmatory HCV PCR and genotyping (Reference Lab Procedures section). The confirmed HCV-positive PWID came from several different sources and were linked to centralized MOH treatment programs through physical tracing by peer educators for further evaluation.

Participants who were HCV PCR-positive underwent clinical review before treatment including; a physical examination for any signs and symptoms of advanced liver disease (ascites and stigmata of cirrhosis), medical history (mental health, TB symptoms, alcohol use, medication review), and the following blood tests to determine treatment eligibility: alanine aminotransferase (ALT), aspartate aminotransferase (AST), and full hemogram. These blood tests were used to calculate AST to platelet ratio index (APRI) score to distinguish those with cirrhosis from those without cirrhosis. Participants eligible for treatment with an APRI score ≤ 1 were initiated on ledipasvir-sofosbuvir. Participants with an APRI score above 1 completed additional tests to classify their degree of cirrhosis using the Childs-Pugh score (CPS) (23). These tests included: international normalized ratio (INR), serum albumin, and serum bilirubin. Individuals with compensated cirrhosis based on CPS class A proceeded with treatment while those with decompensated cirrhosis, defined as CPS class B & C were referred to Kenyatta National Hospital (KNH) for an expert review. Additional baseline tests included creatinine, hepatitis B virus (HBV) surface antigen (sAg), and core antibody (cAb), and pregnancy tests for female participants. General health education and counseling were provided on HCV transmission, risk behaviors, treatment model, and duration at all visits.

### Laboratory procedures

During both screening and SVR-12 visits, a single blood draw of 20 mL was drawn and separated in 2 tubes; 10 mL EDTA purple top and 5 mL SST yellow top vacutainer tubes. Samples were transported daily by a motorcycle courier to (1) the University of Nairobi Pediatric Research Laboratory for the sites in Nairobi and (2) the Malindi sub-county hospital laboratory for sites on the coast. HBsAg testing was conducted using QuickProfile™ HBs antigen, Lumiquick rapid test kits and real-time full hemogram were performed at both laboratories. Both plasma and serum were stored at -80C until processed. Samples were shipped to external laboratories for testing including (1) serum-AST, ALT, and creatinine tests performed using Roche-COBAS INTEGRA 400 plus chemistry analyzer at the Partners in Health and Research Development Laboratory in Nairobi and (2) HBcAb –serum and HCV viral load testing performed using ELISA MUREX anti HBc and GeneXpert, respectively, at the Center for Disease Control laboratory in Kisumu. Because ledipasvir-sofosbuvir is only indicated for treatment of HCV genotypes 1 and 4, it was necessary to genotype all patients prior to treatment initiation. Genotypes were available for most patients through their participation in the parent research study from which they were referred for treatment, with genotyping conducted in the KRISP laboratory at the University of KwaZulu-Natal, South Africa. A small number of patient samples were HCV genotyped locally in the Kenya Medical Research Institute (KEMRI) laboratory in Nairobi.

### Clinical management and treatment

**Figure 1:**
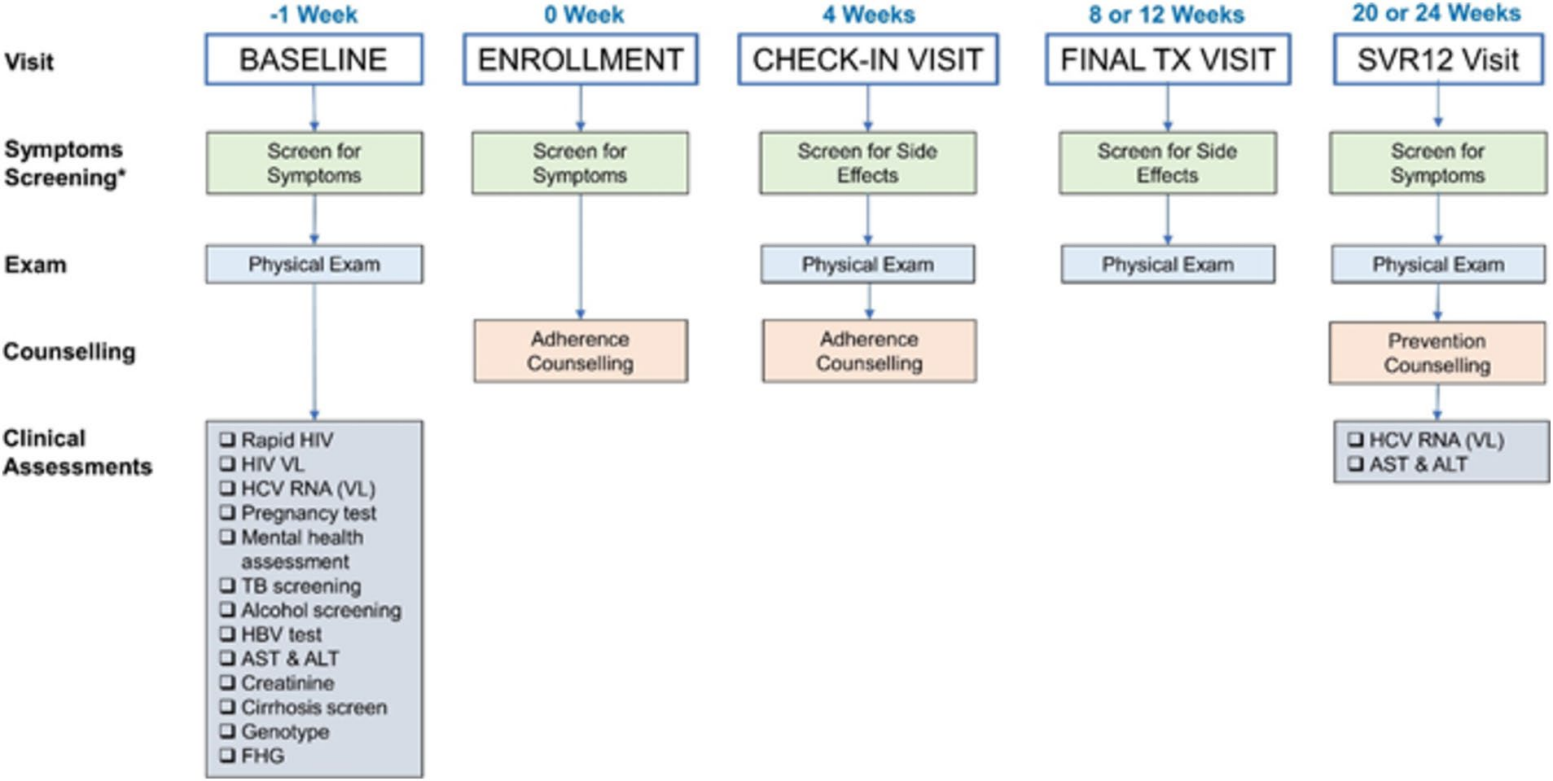
Flow diagram of the treatment process.

Once initiated on treatment, participants traveled every day to the treatment sites to receive their medication dose through DOT. Clinical officers conducted treatment counseling on adherence, dispensed treatment while observing clients taking the drugs, and documented each tablet taken on the DOT clinical record sheet. Participants were treated for different durations depending on their HIV infection status and the extent of their liver damage (Table 2). Participants on methadone received their treatment along with their other medication at methadone clinics, whereas those not on methadone received their treatment at the NSP sites. While on treatment, most participants had follow-up visits with clinical officers at weeks 4, 8, and 12. At each visit their medication was reviewed, they were screened for side effects, a physical exam was conducted, and they were counseled and educated on risk behaviors, harm reduction and treatment adherence. After treatment completion, participants were seen 12 weeks later to assess sustained virologic response (SVR-12). For each participant, the SVR-12 visit comprised of a physical examination, blood tests to assess the HCV viral load, and counseling on HCV re-infection (Figure 1).

**Table 2:** Treatment duration by HIV co-infection status.

Genotype	Combination	HIV Status	Duration
1 and 4	Sofosbuvir/Ledipasvir	HIV-	8 weeks if VL < 6 million IU/mL and no cirrhosis
			12 weeks if VL > 6 million IU/mL and/or with compensated cirrhosis
		HIV+	12 weeks with or without compensated cirrhosis

## Results

Through the treatment project, 142 PWIDs individuals living in Kenya with confirmed active HCV infection were screened for eligibility and willingness to start HCV treatment, of whom 123 (86.6%) were started on HCV treatment using ledipasvir-sofosbuvir. Two individuals (1.4%) were ineligible for treatment due to evidence of advanced liver disease (*n* = 1) or the need for tuberculosis treatment prior the HCV treatment (*n* = 1), and an additional 17 (12.0%) declined to start treatment. Among the 123 individuals who started treatment, 104 (84.6%) completed the full treatment course, 2 (10.5%) died before treatment was completed, and 17 (89.5%) discontinued treatment. Overall, among the 123 individuals who started treatment, a confirmed SVR-12 was achieved by 90 (73.2%) individuals, 19 (15.5%) were confirmed to be viremic at the SVR-12 visit, 3 (2.4%) died before the SVR12 visit, and 11 (9.9%) were lost to follow-up. Future results dissemination will provide details on the treatment outcomes.

### Challenges and lessons learned

Throughout the implementation of this treatment project, challenges that are common barriers to the scale-up of HCV treatment in resource-limited settings were encountered. Financial obstacles were a major concern for most PWID, thus limiting their ability to engage in care. This included daily transport fees to get to and from the dispensing point. Given competing needs (e.g., looking for income for food and shelter), spending limited income on transport to seek health services was a barrier that could have affected treatment adherence; an issue that has been documented in similar settings (10, 24, 25). Additionally, there were privacy concerns at the MAT centers since dispensing windows at MAT centers were overcrowded, and sound barriers were not effective. For these reasons, some PWID enrolled in MAT chose to take their HCV treatment at the NSP sites, which could have added transportation costs and logistical implications that complicated their adherence.

Limited laboratory capacity to conduct screening and confirmatory tests resulted in having the tests conducted at a small number of identified research or private laboratories. The cost of required tests (HCV genotype, HCV viral load and/or PCR) was high at these facilities, which affected the ability of the team to deliver timely diagnosis and treatment, a challenge that has been documented in comparable settings (1, 22, 26). Considering that the predominant genotypes in East Africa are 1a and 4a, simplified treatment options have been advocated through the use of HCV pangenotypic regimes that do not require genotyping and need fewer other lab tests for treatment initiation. These regimens may lessen the cost of treatment, but confirmatory PCR or viral load testing will still be required (25, 27). Frequent incarcerations of PWID interrupted the treatment process for some participants. Most of the incarcerated participants were sent to correctional facilities that were far away from the treatment centers, which made it hard to link the institutions with the health facilities. However, incarcerated clients on methadone were able to access services since they were escorted daily by the prison wardens to the MAT programs to receive their methadone and HCV treatment.

During the implementation of this project, we also noted that there were few available options for clients who required additional testing to identify cases of cirrhosis. The required tests were available in selected private facilities and at high costs that the participants could not afford. While Kenya has a National Health Insurance Fund scheme (NHIF) that supports some of these costs for its members, the majority of PWID are not covered by the NHIF. There is an ongoing effort to register more PWID for NHIF at the NSP and MAT centers. For participants with comorbidities such as active tuberculosis (TB) infection, there were additional challenges initiating HCV treatment due to the need to avoid harmful drug–drug interactions. These participants had to complete their TB treatment first before being initiated on HCV treatment, which might have delayed or complicated their treatment.

Despite these challenges, our experience delivering HCV care during the global COVID-19 pandemic demonstrated that it is possible to provide HCV testing, treatment, and management services at multiple care delivery centers; an important step to improving access to care, especially for key populations (10). A peer-led approach fostered an environment that reduced stigma around HCV through educational sessions, supporting tracing and treatment adherence. Following recent health reforms in Kenya, as NHIF transitions to the Social Health Insurance Fund, this will enhance equity in access to care and remove barriers to accessing healthcare services, especially for PWID who face financial constraints in accessing care (28). Peer educators also acted as a vital link to the facility health staff (clinical officers) in reporting any medication side effects or other issues experienced by the participants in the community.

The project had a limitation: the choice of peer educators may have introduced a selection bias as they were likely to influence the follow-up activities and continued participation of clients in the treatment project. We felt it would be pragmatic to allow the attached institution to select and recommend the peer educators to work with. The peer educators are already working with the rehabilitation institution as community health liaisons to engage PWIDs in HIV, and HCV prevention services.

In conclusion, HCV is a major health threat and treatment remains out of reach for many PWID in Kenya and globally. To meet WHO targets, it is crucial to address barriers throughout the HCV cascade of care, including access to diagnostic and staging tests, access to treatment regimens in a patient-centered integrated care model within the harm reduction organization, and support for people living with HCV to improve their engagement in care is imperative to HCV elimination (22, 25). The release of the National hepatitis guideline in Kenya is a milestone in addressing this growing public health issue (29). Future findings will describe the treatment outcomes of PWID who were treated through this program.

## Data Availability

The original contributions presented in the study are included in the article/supplementary material, further inquiries can be directed to the corresponding author.
